# Clinical presentation and diagnostic evaluation of melanoma in situ using line-field confocal optical coherence tomography

**DOI:** 10.1016/j.jdcr.2025.03.009

**Published:** 2025-03-25

**Authors:** Gabriela Pinero-Crespo, Nyra Taylor, Meredith Thomley, Eugene Sanik, Thomas Beachkofsky

**Affiliations:** a University of South Florida Morsani College of Medicine, Tampa, Florida; b Department of Dermatology and Cutaneous Surgery, University of South Florida Morsani College of Medicine, Tampa, Florida; c Dermatology Service, James A. Haley Veterans Hospital, Tampa, Florida

**Keywords:** LC-OCT, melanoma in situ

## Introduction

Melanoma is an aggressive and potentially deadly skin cancer. Prognosis depends on tumor staging, including depth of invasion, lymph node involvement, and distant metastasis.^1^ Melanoma in situ (MIS) represents an early stage confined to the epidermis.^2^ Skin cancer screening involves a total body skin exam supplemented with dermoscopy by trained providers.^1^, ^2^, ^3^ Skin biopsy with histopathology remains the diagnostic gold standard,^3^ though emerging noninvasive diagnostic tools exist.

Recent developments in noninvasive skin imaging aim to provide real-time diagnoses, reduce unnecessary biopsies, and detect malignancies earlier.^3^ Line-field confocal optical coherence tomography (LC-OCT) is a novel modality allowing in vivo, 3-dimensional visualization of skin diseases to aid in clinical diagnoses and decision-making.^3^ Unlike conventional OCT, with deeper penetration (1-2 mm) but moderate resolution, or reflectance confocal microscopy, with high resolution but limited depth (∼0.2 mm), LC-OCT achieves high cellular resolution comparable to reflectance confocal microscopy while extending the penetration depth to 0.5 mm^3^. This combination visualizes fine cellular structures while maintaining sufficient depth, making LC-OCT particularly valuable for the early assessment of melanoma and other skin cancers. Studies show that LC-OCT can increase the diagnostic accuracy of clinicians evaluating melanocytic and nonmelanocytic skin cancers, at times nearing that of histopathology.^4^

This case report details the presentation of a patient with MIS identified by LC-OCT and corroborated by dermoscopy and histopathology.

## Imaging

**Fig 1:**
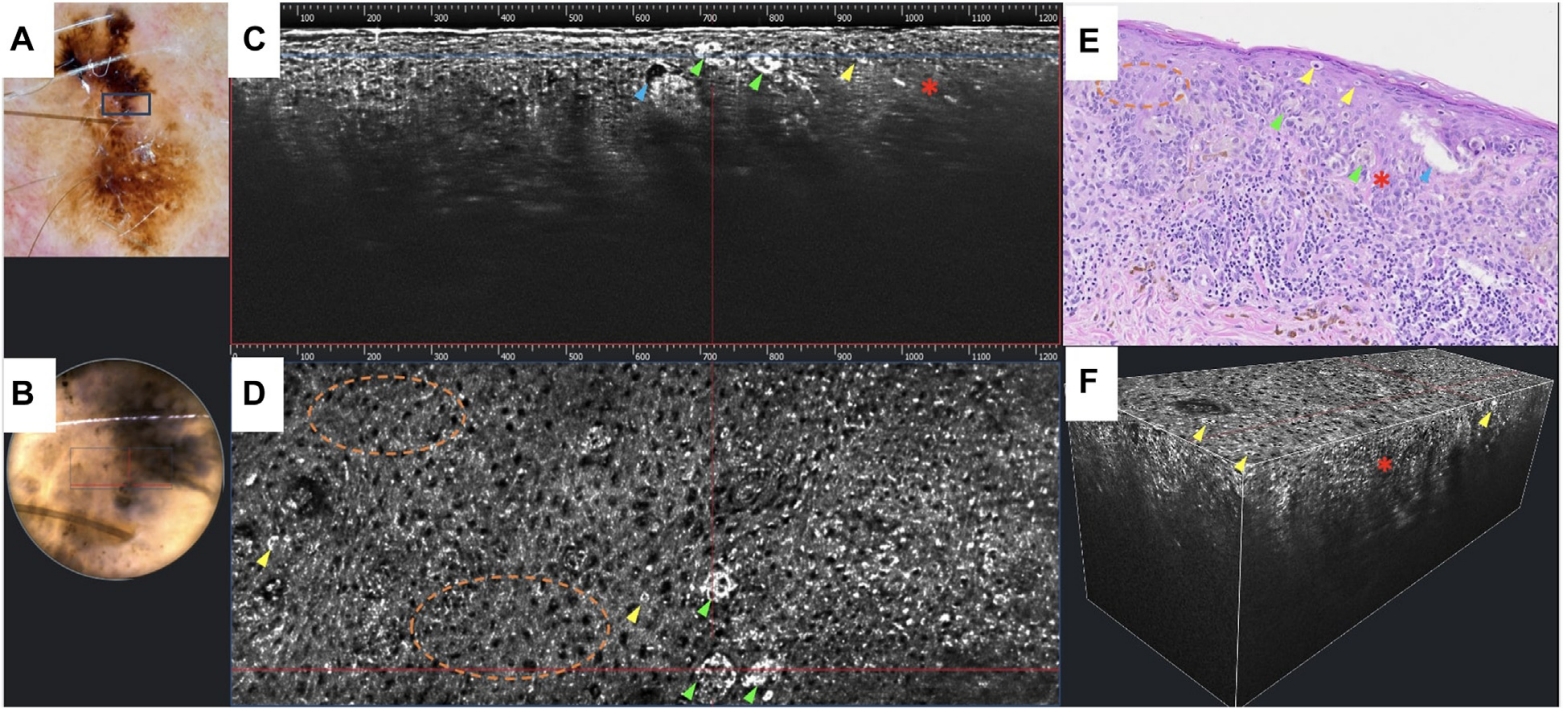
**A,** Melanoma in situ (MIS) dermoscopy image of the right neck. **B,** Real-time dermoscopy image on LC-OCT device. *Blue rectangle* representing the LC-OCT field of view, *red* crosshairs representing correlation between pigment globule and LC-OCT findings. **C,** Cross-sectional 2-D view of LC-OCT. **D,** Top-down 2-D view of LC-OCT. **E,** Histological correlation. **F,** 3-D LC-OCT reconstruction. Key: Irregular honeycomb pattern of keratinocytes (*orange dashed circles*), clefting (*blue arrows*), clusters of pagetoid and atypical cells (*green arrows*), individual pagetoid cells (*yellow arrows*), and disrupted dermal-epidermal junction (*red stars*). *LC-OCT*, Line-field confocal optical coherence tomography.

A 54-year-old male presented with a new pigmented lesion on the right neck. Clinical examination revealed a 7 × 5 mm irregularly pigmented papule without associated lymphadenopathy. Dermoscopy showed an asymmetric multicomponent pattern with atypical dots and globules, an irregular pigment network and blotches, a negative network, focal streaks, and peppering regression structures (Fig 1, *A*). LC-OCT demonstrated highly refractile irregular melanocytic nests, irregular honeycomb pattern, and pagetoid spread representing melanocytes invading the upper epidermis (Fig 1, *C*, *D*, *F*). The lesion was biopsied under suspicion of melanoma.

**Fig 2:**
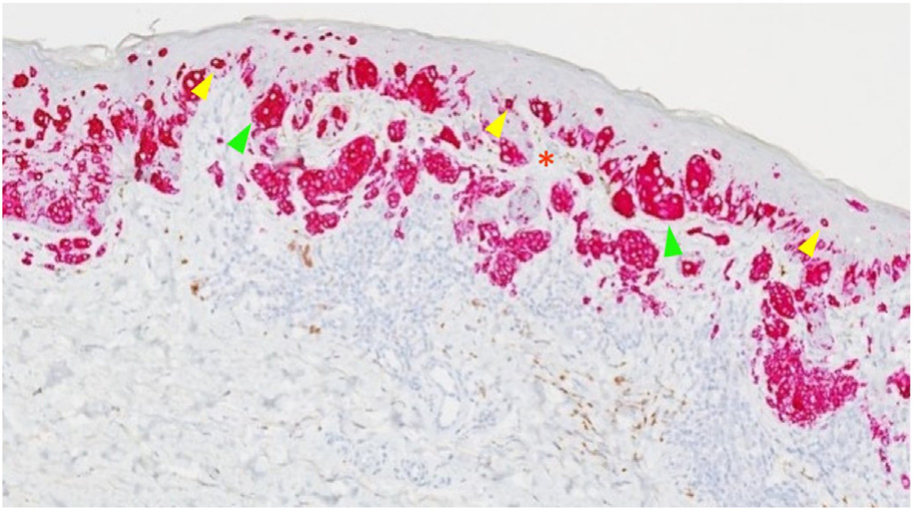
Melanoma in situ histology image with immunohistochemical stain Mart-1 positivity in the cytoplasm of melanocytes. Key: Clusters of pagetoid and atypical cells (*green arrows*), individual pagetoid cells (*yellow arrows*), and disrupted dermal-epidermal junction (*red stars*).

Histopathology confirmed the diagnosis of MIS with marked follicular extension, characterized by confluent growth of atypical melanocytes along the basal epidermis and significant pagetoid scatter extending into the follicular epithelium (Fig 1, *E*). Immunohistochemical staining revealed strong PRAME (Preferentially expressed Antigen in Melanoma) positivity, along with Mart-1 (Fig 2) and Sox-10, supporting the diagnosis of MIS. No true dermal invasion was observed. The lesion was excised with 5 mm margins, and postexcision pathology showed focal residual atypical melanocytes at the junction, with negative margins.

## Discussion

This case highlights the utility of LC-OCT for real-time diagnosis and early detection of melanoma, aiding in clinical decision-making and treatment planning. Perez-Anker et al have proposed melanoma diagnostic criteria for LC-OCT, including features like abundant pagetoid cells, irregular epidermal architecture, clefting, and disrupted dermal-epidermal junction, which were all present in this case.^5^ These architectural criteria are strongly associated with malignancy given their absence in benign nevi.^5^ LC-OCT imaging showed excellent correlation with histopathology by displaying these same diagnostic features.

While LC-OCT provides excellent visualization of superficial structures, its penetration depth is limited, restricting its ability to assess deeper invasion. Thus, histopathologic examination remains essential for complete staging.^3^^,^^5^ Further research is needed to evaluate the full diagnostic capabilities of LC-OCT, particularly its role in distinguishing MIS from invasive melanoma.

## Conflicts of interest

None disclosed.
